# Serious postoperative complications and reoperation after carpal tunnel decompression surgery in England: a nationwide cohort analysis

**DOI:** 10.1016/S2665-9913(20)30238-1

**Published:** 2020-09-30

**Authors:** Jennifer C E Lane, Richard S Craig, Jonathan L Rees, Matthew D Gardiner, Jane Green, Daniel Prieto-Alhambra, Dominic Furniss

**Affiliations:** a Oxford National Institute for Health Research Musculoskeletal Biomedical Research Unit, Nuffield Department of Orthopaedics, Rheumatology, and Musculoskeletal Sciences, Nuffield Orthopaedic Centre, University of Oxford, Oxford, UK; b Kennedy Institute, Nuffield Department of Orthopaedics, Rheumatology, and Musculoskeletal Sciences, Nuffield Orthopaedic Centre, University of Oxford, Oxford, UK; c Department of Plastic and Reconstructive Surgery, Nuffield Orthopaedic Centre, University of Oxford, Oxford, UK; d Frimley Health National Health Service Foundation Trust, Wexham Park Hospital, Slough, UK; e Cancer Epidemiology Unit, Nuffield Department of Population Health, University of Oxford, Oxford, UK

## Abstract

**Background:**

Carpal tunnel decompression surgery to treat carpal tunnel syndrome is a common procedure, yet data on safety and effectiveness of the operation in the general population remain scarce. We aimed to estimate the incidence of reoperation and serious postoperative complications (requiring admission to hospital or further surgery) following carpal tunnel decompression in routine clinical practice and to identify the patient factors associated with these adverse outcomes.

**Methods:**

We did a nationwide cohort analysis including all carpal tunnel decompression surgeries in patients aged 18 years or older, done in the National Health Service in England between April 1, 1998, and March 31, 2017, using the Hospital Episode Statistics dataset linked to mortality records. Patients were followed-up until death or until the end of the study (March 31, 2017). Primary outcomes were the overall incidence of carpal tunnel decompression reoperation and serious postoperative complications (surgical site infection or dehiscence, or neurovascular or tendon injury, requiring admission to hospital or further surgery) within 30 days and 90 days after surgery. Multivariable Cox regression analysis was used to identify factors influencing complications and reoperation, and the Fine and Gray method was used to adjust for the competing risk of mortality. This study is registered with ClinicalTrials.gov, NCT03573765.

**Findings:**

855 832 carpal tunnel decompression surgeries were done between April 1, 1998, and March 31, 2017 (incidence rate 1·10 per 1000 person-years [95% CI 1·02–1·17]). 29 288 procedures (3·42%) led to carpal tunnel decompression reoperation (incidence rate 3·18 per 1000 person-years [95% CI 3·12–3·23]). Of the 855 832 initial surgeries, 620 procedures (0·070% [95% CI 0·067–0·078]) led to a serious complication within 30 days after surgery, and 698 procedures (0·082% [0·076–0·088]) within 90 days. Local complications within 90 days after surgery were associated with male sex (adjusted hazard ratio 2·32 [95% CI 1·74–3·09]) and age category 18–29 years (2·25 [1·10–4·62]). Male sex (adjusted subhazard ratio 1·09 [95% CI 1·06–1·13]), old age (>80 years *vs* 50–59 years: 1·09 [1·03–1·15]), and greater levels of comorbidity (Charlson score ≥5 *vs* 0: 1·25 [1·19–1·32]) and socioeconomic deprivation (most deprived 10% *vs* least deprived 10%: 1·18 [1·10–1·27]) were associated with increased reoperation risk.

**Interpretation:**

To our knowledge, this is the largest national study on carpal tunnel decompression to date, providing strong evidence on serious postoperative complication and reoperation rates. Carpal tunnel decompression appears to be a safe operation in most patients, with an overall serious complication rate (requiring admission to hospital or further surgery) of less than 0·1%.

**Funding:**

Versus Arthritis; Medical Research Council; Royal College of Surgeons of England and National Joint Registry research fellowship; University of Oxford; National Institute for Health Research; and National Institute for Health Research Biomedical Research Centre, Oxford.

## Introduction

Carpal tunnel syndrome is the most common peripheral entrapment neuropathy, caused by compression of the median nerve within the carpal tunnel at the level of the wrist. Estimates of prevalence range between 5% and 10%, with patients presenting to both primary care physicians and secondary care specialists.^1^, ^2^ In the USA, symptomatic carpal tunnel syndrome is reported within government statistics as being the second leading cause of prolonged workplace absence.^3^ Symptoms include pain in the wrist, tingling and numbness in the median nerve distribution, and in severe cases, thumb weakness, leading to functional disability in the hand.^4^

Initial management is non-surgical, but many patients require surgery to decompress the carpal tunnel and preserve hand function.^2^, ^5^ Carpal tunnel decompression surgery is widely undertaken but poorly evaluated, and estimates of complication rates vary widely.^6^, ^7^, ^8^ Results highlighted by the Cochrane collaboration have shown that less than 3000 hands have ever been included in any randomised clinical trial involving carpal tunnel surgery, with the majority of trials exploring the role of endoscopic surgery, a procedure that is rarely done in the UK.^8^, ^9^ Although small observational studies have been able to investigate the risks of some postoperative infections and nerve injuries, the risk and incidence of reoperation, and rarer complications such as deep infection, have been poorly defined because of limitations in coding systems, and the difficulty in enabling full longitudinal follow-up of patients over time within multiple private health-care systems, especially those that do not link to official mortality records.^10^, ^11^, ^12^, ^13^

**Research in context**

**Evidence before this study**

We searched PubMed, EMBASE, PsychINFO, Allied and Complementary Medicine Database, Cumulative Index to Nursing and Allied Health Literature, and Web of Science on August 8, 2019, using the keywords “median neuropathy”, “carpal tunnel syndrome”, “nerve entrapment” or “entrapment neuropathy”, and “hand” or “wrist”, with no language or date restrictions. A Cochrane review published in 2014 comparing endoscopic and open carpal tunnel decompression included the results from 26 studies reporting complications following surgery. Meta-analysis found 0·9% of patients had major complications (including nerve, vascular, or tendon injury) following open decompression and 10·2% had minor complications (including pain, infection, or scar disorders), meaning there was an overall complication rate of 8·7%. The reoperation rate was extracted from ten studies and reported as 2·5%. The review noted a high risk of bias, and the evidence was deemed to be low quality. Three retrospective reviews of US insurance claims datasets or hospital databases for postoperative complication rates have been published, but without analysis of reoperation rates. Although there have been studies in general populations of the incidence of surgery, there is a paucity of evidence on the rate of complications in these populations. Devana and colleagues compared the trends in complications within 90 days of surgery following endoscopic and open carpal tunnel decompression. For open decompression, the study found a postoperative infection rate of 7·97 per 1000 person-years, wound dehiscence rate of 2·87 per 1000 person-years, and median nerve injury rates of 1·69 (Medicare dataset) and 3·72 (Humana dataset) per 1000 person-years. 0·1% of patients required reoperation or readmission within 30 days of surgery in a single-centre review by Goodman and colleagues. Werner and colleagues focused solely on postoperative infection rates in the Medicare dataset after open decompression, and found an infection rate of 0·32%. Infection risk was associated with younger age, male sex, obesity, tobacco smoking, and greater levels of comorbidities. To our knowledge, there has been no longitudinal population-based study that has determined the long-term incidence of reoperation, or risk factors associated with both reoperation and complications, after carpal tunnel decompression surgery.

**Added value of this study**

We provide new evidence of the incidence of reoperation and local complications from a nationwide publicly funded health service with long follow-up in an unselected patient group. Adverse events recorded in a hospital admissions database were uncommon, with a 90-day local serious complication rate of 0·082%, and a reoperation incidence rate of three per 1000 person-years. Reoperation mostly occurred within the first postoperative year, with a median time to reoperation of 351 days. Excess risk of reoperation was associated with male sex, old age (>80 years *vs* 50–59 years), greater levels of comorbidities, and socioeconomic deprivation, after adjustment for the competing risk of death. By contrast, local serious complications in the first 90 days after surgery were more likely in men and younger patients aged 18–29 years but were not associated with medical comorbidity as previously described.

**Implications of all the available evidence**

Carpal tunnel decompression surgery, as practiced in the National Health Service in England, appears to be safe and has a low incidence of reoperation and local serious complications. Reoperation was more common in older patients, those with more comorbidities, and those with increased social deprivation; whereas, early local serious complications were most often seen in younger male patients. This evidence should inform health-care commissioners and clinicians about the role of decompression surgery within the treatment of carpal tunnel syndrome, and guide personalised shared decision making.

Increasingly, evidence from routinely collected data is used to evaluate health-care interventions in the general population.^14^ Outcome data from real world practice enables improved counselling of patients both before referral by primary care physicians, and before surgery. The identification of markers for greater risks of complications or reoperation allows personalised shared decision making. Similarly, as national health technology assessments become increasingly focused on evidence from routine clinical practice, providing evidence for the outcomes following a frequently undertaken procedure within clinical practice is important.^15^

We aimed primarily to estimate the risks of serious postoperative complications (requiring admission to hospital or further surgery) and reoperation following carpal tunnel decompression surgery in adults in the National Health Service (NHS) in England. Our secondary aim was to identify risk factors associated with these adverse outcomes, to improve counselling of those patients at greater risk.

## Methods

### Study design and participants

We analysed a nationwide cohort including all patients aged 18 years or older undergoing carpal tunnel decompression surgery in the NHS in England between April 1, 1998, and March 31, 2017, with follow up until death or until the end of the study (March 31, 2017).

Minimum follow-up was 1 day after surgery, because of the authors' clinical experience that reoperation could be required within the first 24 h after the initial surgery because of an incomplete nerve release combined with acute swelling. Follow-up continued until death to maximise the duration of postoperative longitudinal follow-up available, which was the advantage of using this study design and observational data.

All carpal tunnel decompression operations associated with trauma in the same episode were excluded, because acute carpal tunnel syndrome in the context of trauma has a different pathophysiology. This study was approved by the University of Oxford Research Services Clinical Trials Research Group (ID 12787), and the NHS Data Access Advisory Group. Studies using non-identifiable records from Hospital Episode Statistics are exempt from research ethics committee approval, and patients had the right to request that their data was not released by NHS Digital for use by researchers (a type 2 opt-out).

### Data source

A bespoke extract from Hospital Episode Statistics Admitted Patient Care data was generated, using individual-level patient data, containing all episodes of NHS patient care in the entire dataset for all individuals with an index (first recorded) carpal tunnel decompression episode.^16^ The extract contained all episodes of care remunerated by the NHS in England, including episodes in all NHS hospitals in addition to independent providers (ie, private hospitals undertaking procedures for patients under the NHS). The Hospital Episode Statistics Admitted Patient Care dataset is able to link together all episodes of NHS care a patient has in England through an individual NHS number, allowing longitudinal follow-up and identification of complications and reoperation done by any NHS health-care provider, even if this subsequent event was not undertaken by the same provider as the index event. Before pseudonymisation, NHS Digital also linked the Hospital Episode Statistics extract to the Office for National Statistics national mortality dataset to identify cause and date of death.^17^ The NHS covers the vast majority of health-care provision in England, with only 11% of the population estimated to hold private health insurance, and only 13% of all elective surgery being privately funded outside of the NHS.^18^ Duplication can occur when episodes of care span over two financial years, and therefore the dataset was cleaned to ensure all episodes were non-duplicates.

### Exposures and outcomes

Exposures and outcomes were defined using previously validated Office of Population Censuses and Surveys Classification of Interventions and Procedures (OPCS) 4.7 and International Classification of Diseases 10 (ICD-10) codes, and comorbidities were defined using ICD-10 codes (appendix p 1).^19^, ^20^, ^21^ Carpal tunnel decompression surgery was defined using OPCS codes A65.1 or A69.2, A65.8 with Z09.2, or A65.9 with Z09.2, in association with ICD-10 code G56.0. Known risk factors for carpal tunnel syndrome were also defined using ICD-10 codes including: past medical history of diabetes, hand osteoarthritis, gout, wrist fracture, rheumatoid arthritis, obesity, and hypothyroidism.^5^, ^22^ Patients were considered to have a past medical history of a condition if a code associated with the condition was found within the hospital episode for carpal tunnel decompression surgery, or any previous hospital episode. Index of multiple deprivation (a government-generated score of relative deprivation based on geographical location within England), Charlson comorbidity index, and ethnicity were also considered as exposures that might be associated with the incidence of reoperation and adverse events.^23^, ^24^ The Charlson comorbidity index was developed to assess the global level of comorbidity of a patient to predict mortality. We used the modified version developed in 2011, updated to reassess the relative burden of disease and risk of mortality associated with each included condition.

Serious complications were defined as surgical site infection or dehiscence requiring surgery or admission to hospital and neurovascular or tendon injury that resulted in a further episode of care within the dataset and that occurred on the same hand as the index procedure. Complications were also defined using OPCS and ICD-10 codes (appendix p 1). Carpal tunnel decompression reoperation was defined as an OPCS code for revision decompression and a previous code for primary decompression on the same hand; two primary decompression codes in the same patient on the same hand; or three or more primary decompression codes in the same individual. We considered that decompression surgery coded on three separate dates, without laterality defined, identified patients who had undergone revision on either hand. As the laterality was not defined for these patients, we included them only to identify the total burden of reoperation in the cohort, as linkage between the index surgery and the reoperation was not possible because of missing laterality. These were defined as an unpaired revision carpal tunnel decompression surgery within our data management process. For survival analysis and reoperation incidence rate, where time to event is crucial, only those patients with defined paired laterality in two decompressions were included and defined as paired revisions within our data management process.

### Outcomes

We determined complication rates within 30 or 90 days of surgery to align with the national outcome framework in England that enables comparison of outcomes following all procedures within the NHS.^25^

### Statistical analysis

We calculated age-specific and sex-specific incidence rates of surgery using Office for National Statistics mid-year population estimates.^26^ All complications were calculated as a proportion of the sample with 95% CI. Incomplete records consisted of only 0·72% of cases, with missing data in the fields of age, sex, ethnicity, and index of multiple deprivation deciles, and were assumed to be missing completely at random. We therefore did a complete case analysis and did not employ any imputation. Laterality was missing in 3·8% of cases; comparison of demographics of those with and without laterality present within their records demonstrated that the patients were comparable (appendix pp 1–2). We did a Kaplan-Meier analysis to identify the trend in time to reoperation. We identified factors associated with time to reoperation using a Fine and Gray model to produce crude subhazard ratios and a final adjusted model, to account for the competing risk of mortality.^27^ The model was adjusted for age, sex, social deprivation (as defined by index of multiple deprivation), and overall level of comorbidity (as defined by the modified Charlson score).^24^ Risks of complications within 90 days of surgery were assessed using Cox proportional hazard modelling only, but again adjusted for age, sex, social deprivation, and overall level of comorbidity. The proportional hazards assumption was tested using Schoenfeld residuals. Age was grouped into 10-year categories because of the non-linear relationship of age with adverse outcome, while attempting to keep a sufficient number of patients within each group, and to produce the minimum number of categories to prevent loss of power within the model. The category containing the median age (50–59 years) was used as the reference level. We analysed the trend in the number of surgeries over time, which was not considered to significantly differ, and therefore stratification by time was not added to the model. This concurs with the clinical rationale of the authors that over the time period of the study, there has not been a substantial change in surgical technique used within the NHS, with open carpal tunnel decompression remaining as the primary technique. Statistical analysis was done using STATA version 15.1. This study is registered with ClinicalTrials.gov, NCT03573765.

### Role of the funding source

The funders of the study had no role in study design, data collection, data analysis, data interpretation, or writing of the report. All authors had access to all study data and had final responsibility for the decision to submit for publication.

## Results

**Figure 1:**
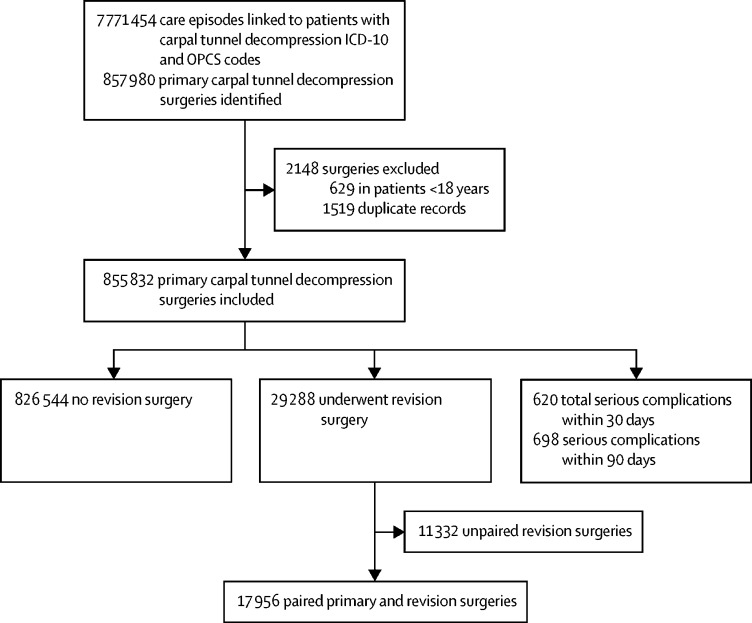
Study profile ICD-10=International Classification of Diseases 10. OPCS=Office of Population Censuses and Surveys Classification of Interventions and Procedures.

During the study period from April 1, 1998, to March 31, 2017, 855 832 primary carpal tunnel decompression operations were done on 665 090 individuals in hospitals in England within the NHS (figure 1, table 1, appendix pp 2–3); equivalent to a primary decompression surgery incidence rate of 1·10 per 1000 person-years (95% CI 1·02–1·17), and a sex-specific incidence rate of 0·73 per 1000 person-years (0·73–0·74) in men and 1·47 per 1000 person-years (1·47–1·48) in women.

**Table 1:** Patient demographics

		**Primary carpal tunnel decompression (n=855 832)**	Revision or complication*(n=29 936)
Sex			
	Female	581 645 (67·96%)	19 333 (64·58%)
	Male	273 845 (32·00%)	10 600 (35·41%)
	Missing data	342 (0·040%)	3 (0·010%)
Age, years		57·9 (15·6)	59·7 (15·6)
IMD decile			
	10 (least deprived)	76 234 (8·91%)	2481 (8·29%)
	9	85 801 (10·03%)	2749 (9·18%)
	8	87 873 (10·27%)	2992 (9·99%)
	7	91 842 (10·73%)	3187 (10·65%)
	6	90 257 (10·55%)	3129 (10·45%)
	5	81 535 (9·53%)	3071 (10·26%)
	4	83 448 (9·75%)	3047 (10·18%)
	3	85 702 (10·01%)	3021 (10·09%)
	2	89 949 (10·51%)	3137 (10·48%)
	1 (most deprived)	77 114 (9·01%)	2930 (9·79%)
	Missing data	6077 (0·71%)	192 (0·64%)
Ethnic group			
	Any white background	641 678 (74·98%)	24 375 (81·42%)
	Any Asian background	19 295 (2·25%)	701 (2·34%)
	Any Black background	10 645 (1·24%)	472 (1·58%)
	Any mixed background	2679 (0·31%)	118 (0·39%)
	Chinese	1181 (0·14%)	25 (0·084%)
	Any other ethnic group	8268 (0·97%)	262 (0·88%)
	Not known	172 86 (20·11%)	3983 (13·31%)
Charlson comorbidity index			
	0	389 351 (45·49%)	11 585 (38·70%)
	1	179 437 (20·97%)	6908 (23·08%)
	2	97 719 (11·42%)	3709 (12·39%)
	3–4	98 781 (11·54%)	4027 (13·45%)
	≥5	90 544 (10·58%)	3707 (12·38%)
	Missing data	0	0

Data are n (%) or mean (SD). IMD=index of multiple deprivation.

* Complications included neurovascular injury, tendon injury, wound infection, and wound dehiscence, identified up to 90 days after primary surgery. 50 patients sustained more than one complication or had more than one reoperation within the study period.

**Figure 2:**
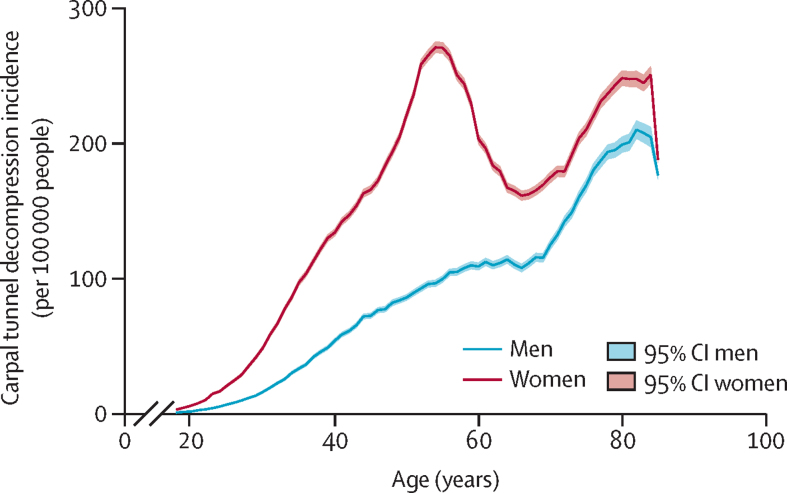
Incidence of primary carpal tunnel decompression by age and sex

For primary carpal tunnel decompression, 581 645 (68·0%) of 855 832 operations were on female patients, and the overall median age at surgery was 57·1 years (IQR 46·9–70·7). There was a large peak in incidence of decompression operations in the perimenopausal age range in women, with no equivalent peak seen in men (figure 2).

The median follow-up time for all procedures was 7·53 years (3·65–12·02). 3665 procedures (0·43%) had less than 30 days of follow-up without an event, because the surgery was done during March, 2017. 12 020 procedures (1·40%) had less than 90 days of follow-up without an event, because the surgery was done between Jan 1 and March 31, 2017. 126 606 cases (14·79%) died during the follow-up period without sustaining a serious complication or reoperation. The median length of follow-up for those who died was 6·36 years (IQR 3·42–9·80), and therefore is similar to the length of follow-up for those who survived to the end of the study period, and sufficient time to identify a short-term complication or reoperation.

Overall rates of serious complications were very low (table 2); local serious complications occurred after 620 (0·070%; 95% CI 0·067–0·078) of 855 832 procedures within 30 days after surgery, and after 698 procedures (0·082%; 0·076–0·088) within 90 days. Of all serious complications, wound dehiscence (282 events [0·032%; 0·029–0·037]) and tendon injury (285 events [0·033%; 0·030–0·037]) had the highest incidence within 90 days after surgery.

**Table 2:** Serious postoperative complication rates following primary carpal tunnel decompression

	**Total cases, n**	**Percentage of procedures (95% CI)**
**Wound dehiscence**		
Within 30 days	259	0·030% (0·027–0·034)
Within 90 days	282	0·033% (0·029–0·037)
**Wound infection**		
Within 30 days	32	0·0037% (0·0026–0·0053)
Within 90 days	43	0·0050% (0·0037–0·0068)
**Tendon injury**		
Within 30 days	241	0·028% (0·025–0·031)
Within 90 days	285	0·033% (0·030–0·037)
**Neurovascular injury**		
Within 30 days	88	0·010% (0·0083–0·013)
Within 90 days	88	0·010% (0·0083–0·013)
**Any complication**		
Within 30 days	620	0·072% (0·067–0·078)
Within 90 days	698	0·082% (0·076–0·088)

**Figure 3:**
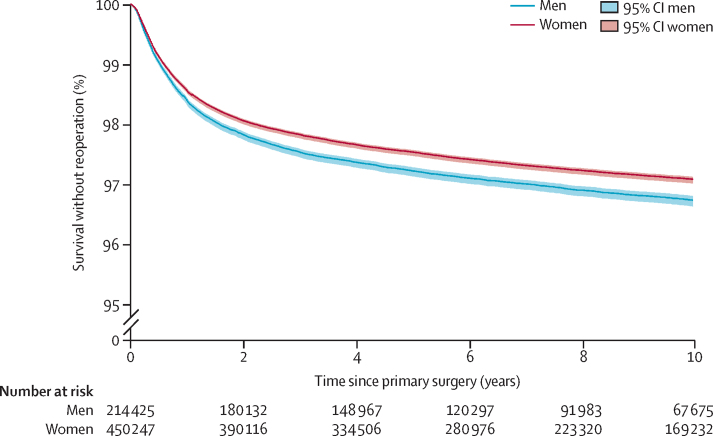
Representative Kaplan-Meier analysis for reoperations within the first 10 years after primary surgery For visual clarity there is a break in the y-axis between 0% and 95%.

29 288 reoperations (3·42%) were done during the study period. The incidence rate for all reoperation surgeries was 3·18 per 1000 person-years (95% CI 3·13–3·22; table 1). There were 17 956 paired primary and reoperation procedures. 18 968 (64·76%) of 29 288 patients who had a reoperation were female, with an overall median age of 60 years (IQR 49–74). The median time to reoperation was 351 days (IQR 144–966), calculated from observed data for each individual, taking into account the individual varying follow-up time from date of the primary surgery to date of the reoperation. Kaplan-Meier analysis provided an estimated median time to reoperation of 331 days (95% CI 322–338), which shows that reoperation predominantly occurred within the first year after surgery, with no further peak in reoperation incidence at any later timepoint (figure 3). The trend in time to reoperation was similar in both sexes, albeit with a greater number of revision surgeries in men than in women.

Male sex was associated with an increased risk of reoperation (subhazard ratio 1·09 [95% CI 1·06–1·13]; appendix p 4). Reoperation was also associated with higher levels of comorbidity; patients with a Charlson score of 2 had an increased relative risk of reoperation compared with patients with a score of 0 (subhazard ratio 1·17 [1·11–1·23]), which increased with a score of 3–4 (1·26 [1·20–1·33]), and with a score of 5 or higher (1·25 [1·19–1·32]). Reoperation risk was consistently increased in those with a Charlson score of 1 or higher (appendix p 4). Old age (>70 years) and increasing deprivation were also associated with an increased risk of reoperation. Patients in the most deprived decile of the deprivation index had a higher risk of reoperation compared with those in the least deprived decile (HR 1·18, 95% CI 1·10–1·27; appendix p 4).

Male sex was also associated with an increased incidence of serious local complications within 90 days after surgery (adjusted hazard ratio 2·32 [95% CI 1·74–3·09]; appendix p 5). In contrast to reoperation, there was no association between local complications and Charlson score or deprivation index. There was an inverse association between serious local complication and age; patients aged 18–29 years had an increased risk of serious local complications within 90 days (adjusted hazard ratio 2·25 [1·10–4·62]; appendix p 5).

## Discussion

This large observational study, using data from a nationwide publicly funded health service, shows that the incidence of carpal tunnel decompression surgery for an unselected, general population-based patient group in the NHS in England is approximately 1 in 1000 people per year. Compared with published studies from other countries, this incidence is low.^1^, ^2^, ^28^ However, as previous studies comparing surgical practices in England for upper limb conditions have shown a lower intervention rate, we consider this difference to reflect changes in clinical practice in England in comparison to other countries, rather than incomplete data capture.^29^, ^30^, ^31^ Carpal tunnel decompression surgery was more common in women than men, especially among women in the perimenopausal age range. Longitudinal follow-up within this dataset enabled us to calculate overall serious complication rates, which show that carpal tunnel decompression is usually a safe operation. We found low rates of reoperation, surgical site infection, and nerve or tendon injury, especially in comparison with rates reported in clinical trials.^8^ Rates of postoperative infection in this NHS cohort are also lower than those described in the US Medicare population.^11^ Although studies have been done in the UK to capture the rate of carpal tunnel syndrome and decompression surgery, to date we have not found a study that reports complication rates including reoperation and other rarer complications in a general population.^2^, ^5^, ^13^

We found that fewer men than women undergo primary carpal tunnel decompression, but they have an increased incidence of reoperation and serious local complications. There could be a variety of reasons for this increased incidence, but we postulate that it could be because of surgery being more technically difficult in a larger hand as there is a greater amount of subcutaneous tissue in the operative field, reducing surgical exposure of the transverse carpal ligament. There is also evidence in the literature of a different pathophysiology of carpal tunnel syndrome in women that is hormonally driven, and might lead to disparity in outcome between the sexes.^22^ Increased risk of reoperation in those with more comorbidities and increased risk of local complications in younger men have been previously reported.^13^ Increased risk of reoperation has also been reported in those who return to manual labour, repetitive tasks, or using vibrating tools, which could provide a reason for the increase in reoperation risk in those in lower-income socioeconomic groups, but is outside the scope of this study.^32^ It is important to note however that in this study, the overall serious complication rates are very low, and therefore the increased incidence in men might not represent a clinically relevant increase in risk. Therefore, our main conclusion from this study remains that carpal tunnel decompression surgery appears safe for most patients.

The strength of this study lies in its ability to reflect the volume of surgery undertaken in a public health-care system across an entire country, including patients from extremes of the age distribution, from all classes of deprivation, and with coexisting comorbidities. Using data from a universal public provider of care linked to mortality data enables long-term follow-up across hospital providers that is not possible with private insurance datasets, and this study is reassuring regarding the safety of carpal tunnel decompression surgery. Furthermore, the linkage to all NHS hospital episodes for each individual provides the best available estimate of complications and reoperation, as the event is captured even if a patient transfers between hospitals. The availability of information for each hospital episode also allowed us to focus on chronic carpal tunnel syndrome, excluding acute cases with a different pathophysiology, which is not possible in other datasets. The long follow-up period available enabled the long-term risk of reoperation to be determined. The long follow-up and universal nature of NHS data provided in the Hospital Episode Statistics dataset provides nuance to identifying the population included, and the potential risk of loss to follow-up. All patients remain in follow-up until emigration, death, transfer of care to the private sector, or they request their data to be removed from NHS datasets. Emigration during the study period is thought to be stable at approximately 300 000–400 000 people per year, and the coverage of private health insurance and elective surgical provision is low in England.^18^, ^33^ As this study has linked coverage to mortality data, we were able to evaluate the precise follow-up period in patients who died before the end of the study period. The follow-up period in these patients was still long on average (median 6·3 years [IQR 3·4–9·8]); nearly the same as in those surviving to the end of the study period, and sufficient for short-term complications and the median time to reoperation seen in the cohort. We did not consider deaths before the end of the study period as a noteworthy source of bias.

Patients can request removal of their data from NHS databases as part of the type 2 opt-out system, which could impact loss to follow-up.^34^ The current proportion of patients requesting removal of their data from NHS Hospital Episode Statistics datasets is approximately 2·6%, and therefore data removal requests are likely to have a small effect on loss to follow-up within our static, bespoke, pseudonymised extract.

Our findings can be used to inform health-care policy, and individual patient decision making when considering surgery for carpal tunnel syndrome. Although the Hospital Episode Statistics dataset only includes patients who have been admitted to secondary care, this set includes all day-case procedures, and as almost all carpal tunnel decompression surgeries are done in secondary care as day-case procedures, there is a reduced risk of referral bias. Clinically, guidelines dictate that surgery in England is only done in more severe cases of carpal tunnel syndrome, and when comparing the demographics of the patients included in our cohort they appear similar to those managed operatively in the USA and to those in studies in UK primary care.^2^, ^35^, ^36^

The Hospital Episode Statistics dataset was originally designed to calculate costs within the NHS rather than for research purposes, and therefore there are limitations of the dataset. Data regarding some risk factors for carpal tunnel decompression or for surgical complications, such as occupation or tobacco smoking status, are not recorded, and others such as obesity are likely to be under-reported. Because of this unrecorded information, especially surrounding occupation, unobserved confounding could occur.

Furthermore, minor complications such as a wound infection treated with antibiotics in primary care are not observed. This means that this study in secondary care will underestimate the rate of all infective complications and other minor complications. However, identification of serious complications is very relevant for patients, by providing information regarding major risks of surgery, and for health-care commissioners, to benchmark health-care providers by the rates of serious complications compared with the national rate within the NHS found in this study. Current NHS commissioning guidelines for carpal tunnel syndrome do not contain data from routine clinical practice surrounding the rate of serious infection or complications after surgery, and therefore this study directly contributes to the current evidence base for clinical care as the NHS moves towards providing individualised outcome data for surgeons and health-care providers. Future studies should extend this research to investigate the rate of infection recorded in primary care, and where information is available, to define infection according to nationally recognised criteria.

An administrative dataset only collects data that contributes to remuneration, and therefore complications that are not attributed to a further admission cannot be assessed using this cohort. In the context of carpal tunnel decompression surgery, complications such as complex regional pain syndrome and scar tenderness are not collected within the Hospital Episode Statistics dataset, and therefore missing data on these complications is a limitation of the study. Similarly, we used two different code combinations to identify nerve injury; that associated with the median nerve itself, and the generic code for nerve injury. This second code does not elaborate further on the particular nerve injured, and therefore in some cases we are unable to differentiate between damage to the median nerve and its palmar cutaneous branch. When considering reoperation, we clinically interpret repeat operations during the first year after the primary surgery as attributable to inadequate initial decompression and therefore persistence of initial symptoms, and the reoperations occurring many years after surgery as being more likely to be because of recurrence. The aim of this study was to determine the rate of and time to reoperation at a population level in routine clinical practice, and therefore further work investigating the reason for reoperation should be done using a different study design.

Outcomes from surgical procedures are unique in that they can be affected by patient, surgeon, hospital, and health-care system-related factors. This study has investigated patient factors associated with outcomes, but future work should focus on the unique interplay of these multiple factors on the outcome from a surgical procedure, when there is further consensus from the statistical community about how to deal with these factors within multilevel modelling techniques.

Routine collection of patient-reported outcome measures have revolutionised other areas of surgery, to enable surgical provision to remain patient focused when undertaking commissioning and health economic assessments. The British Society for Surgery of the Hand has promoted the collection of patient-reported outcome data from a professional perspective, but such collection is not mandatory, and is therefore not truly representative of NHS workload. Although complication rates and rates of reoperation are crucial for informing outcome data, a further cost-effectiveness analysis based on mandatory patient-reported outcome measures collection would be valuable to assess the outcome from the patient perspective and to compare cost-effectiveness of carpal tunnel decompression and other modalities in the treatment of carpal tunnel syndrome.

## Data sharing

This study was carried out in accordance with the NHS Digital Data Sharing Agreement (DARS-NIC-29827-Q8Z7Q). No further data can be made available because of NHS Digital restrictions. Data extracts can be applied for via the NHS Digital data access request service.
