# Supplementary material for: Serious postoperative complications and reoperation after carpal tunnel decompression surgery in England: a nationwide cohort analysis
Source: Lancet Rheumatol. 2020 Sep 30;3(1):e49–57. doi: 10.1016/S2665-9913(20)30238-1 (PMC7762724; doi:10.1016/S2665-9913(20)30238-1)

THE LANCET
Rheumatology

Supplementary appendix

This appendix formed part of the original submission and has been peer reviewed. We post it as supplied by the authors.

Supplement to: Lane JCE, Craig RS, Rees JL, et al. Serious postoperative complications and reoperation after carpal tunnel decompression surgery in England: a nationwide cohort analysis. *Lancet Rheumatol* 2020; published online Sept 30. https://doi.org/10.1016/S2665-9913(20)30238-1.

Supplementary Table 1. OPCS-4.7 and ICD-10 code combinations for identification of CTD cases

CTS OPCS codes			With	ICD-10 code
A65.1	Alone			G56.0
A69.2	Alone			
A65.8	**AND**	Z09.2		
A65.9	**AND**	Z09.2		

Supplementary Table 2.ICD-10 code combinations for identification of CTD covariates

Covariates	ICD-10 code
Diabetes mellitus	E10-14
Obesity	E66
Hypothyroid	E02-03
Hand OA	M18; M15.1, M15.2
Rheumatoid Arthritis	M05
Gout	M10
Wrist/Hand Fracture	S62

Supplementary Table 3. OPCS-4.7 and ICD-10 code combinations for CTD complications

Complications	OPCS code	Timeframe for complication
Wound dehiscence	Z82 + (S60.4 or S42.2 or S42.3 or S42.4)	Within 30 or 90 days
Tendon repair	Z82 + T67	Within 30 or 90 days
Wound debridement	Z82 + (T96.8 or T96.3 or T96.4)	Within 30 or 90 days
Neurovascular injury	A64 + Z09.2 (median nerve repair) or<br>ICD code T81.2	Within 30 days

Supplementary Table 4. Demographics of those with and without laterality

	Primary CTD with laterality Number (%)	Primary CTD without laterality Number (%)
**Sex**		
**Female**	559 661 (68.01)	21 983 (66.79)
**Male**	262 934 (31.95)	10 911 (33.15)
**Missing**	320 (0.04)	22 (0.07)
**Total**	822 915	32 916
**Median age [IQR]**	57.08 [46.91, 70.79]	56.8 [46.98, 69.66]
**Indices of Multiple Deprivation decile**		
**Least deprived 10%**	73 490 (8.93)	2 744 (8.34)
**Less deprived 10-20%**	82 664 (10.05)	3 136 (9.53)
**Less deprived 20-30%**	84 345 (10.25)	3 528 (10.72)
**Less deprived 30-40%**	88 317 (10.73)	3 525 (10.71)
**Less deprived 40-50%**	86 623 (10.53)	3 634 (11.04)
**More deprived 10-20%**	81 535 (9.60)	3 048 (9.26)
**More deprived 20-30%**	80 390 (9.77)	3 058 (9.29)
**More deprived 30-40%**	82 724 (10.05)	2 978 (9.05)
**More deprived 40-50%**	86 570 (10.52)	3 379 (10.27)
**Most deprived 10%**	74 015 (8.99)	3 099 (9.41)
**Missing**	5 290 (0.64)	787 (2.39)
**Total**	822 915	32 916
**Ethnic group**		
**Any white background**	620 047 (75.35)	21 631 (65.71)
**Any Asian background**	18 772 (2.29)	523 (1.59)
**Any Black background**	10 303 (1.26)	342 (1.05)
**Any mixed background**	2600 (0.32)	79 (0.24)
**Chinese**	1 148 (0.14)	33 (0.1)
**Any other ethnic group**	8 027 (0.97)	241 (0.73)

**Not given/not known**	162 018 (19.69)	10 067 (30.58)
**Total**	822 915	32 916
**Charlson index**		
**0**	374 982 (45.57)	14 747 (44.80)
**1**	172 877 (21.01)	6 560 (19.93)
**2**	93 847 (11.40)	3 835 (11.65)
**3-4**	94 701 (11.51)	3 963 (12.04)
**5+**	86 508 (10.55)	3 811 (11.58)
**Missing**	0	0
**Total**	822 915	32 916

Supplementary Table 5. Demographics of patients included

	**Primary CTD Number (%)**	**Revision CTD Number (%)**	**Other Complications[a] Number (%)**
Sex			
**Female**	581 645 (67·96)	18 968 (64·76)	375 (56·48)
**Male**	273 845 (32·00)	10 318 (35·23)	289 (43·52)
**Missing**	342 (0·04)	2 (0·01)	0
**Total**	855 832	29 288	664
**Age category (years)**			
**18-29**	19 042 (2·23)	501 (1·72)	20 (3·01)
**30-39**	87 159 (10·20)	2 513 (8·63)	64 (9·64)
**40-49**	164 434 (19·25)	5 515 (18·94)	117 (17·62)
**50-59**	215 441 (25·22)	6 740 (23·15)	174 (26·20)
**60-69**	145 162 (16·99)	5 240 (18·00)	115 (17·32)
**70-79**	131 493 (15·39)	4 985 (17·12)	104 (15·66)
**>80**	91 465 (10·71)	3 617 (12·42)	70 (10·54)
**Missing**	1636 (0·19)	177 (0·60)	0
**Total**	855 832	29 288	664
**Indices of Multiple Deprivation decile**			
**Least deprived 10%**	76 234 (8·97)	2 416 (8·30)	65 (9·79)
**Less deprived 10-20%**	85 801 (10·10)	2 694 (9·26)	55 (8·28)
**Less deprived 20-30%**	87 873 (10·34)	2 925 (10·05)	69 (10·39)
**Less deprived 30-40%**	91 842 (10·81)	3 115 (10·70)	75 (11·30)
**Less deprived 40-50%**	90 257 (10·62)	3 061 (10·52)	69 (10·39)
**More deprived 10-20%**	81 535 (9·60)	3 006 (10·33)	67 (10·09)
**More deprived 20-30%**	83 448 (9·82)	2 983 (10·25)	65 (9·79)
**More deprived 30-40%**	85 702 (10·09)	2 963 (10·18)	61 (9·19)
**More deprived 40-50%**	89 949 (10·59)	3 062 (10·52)	77 (11·60)
**Most deprived 10%**	77 114 (9·07)	2 876 (9·88)	55 (8·28)
**Missing**	6 077 (0·71)	187 (0·64)	*
**Total**	855 832	29 288	664
**Ethnic group**			
**Any white background**	641 678 (74·97)	23 836 (81·38)	551 (82·99)
**Any Asian background**	19 295 (2·26)	689 (2·35)	13 (1·95)
**Any Black background**	10 645 (1·24)	462 (1·59)	10 (1·50)
**Any mixed background**	2 679 (0·32)	118 (0·41)	*
**Chinese**	1 181 (0·14)	24 (0·08)	*
**Any other ethnic group**	8 268 (0·96)	256 (0·87)	*
**Not known/not given**	172 086 (20·11)	3 904 (13·33)	83 (12.49)
**Total**	855 832	29 288	664
**Charlson index**			
**0**	389 351 (45·50)	11 336 (40·07)	256 (38·55)
**1**	179 437 (21·00)	6 762 (23·08)	149 (22·44)
**2**	97 719 (11·42)	3 617 (12·35)	93 (14·01)
**3-4**	98 781 (11·54)	3 952 (13·49)	77 (11·60)
**5+**	90 544 (10·58)	3 621 (12·36)	89 (13·40)
**Missing**	0	0	0
**Total**	855 832	29 288	664

**Predisposing factors**			
**DM**	50 070 (5·85)	2 305 (7·87)	47 (7·08)
**Obesity**	21 885 (2·56)	1 112 (3·80)	21 (3·16)
**Hypothyroidism**	20 784 (2·43)	883 (3·01)	24 (3·61)
**RA**	1 117 (0·13)	43 (0·15)	*
**Hand OA**	2 388 (0·28)	124 (0·042)	*
**Gout**	4 373 (0·51)	213 (0·73)	8 (1·20)
**Previous wrist fracture**	3 479 (0·40)	114 (0·39)	8 (1·20)

[a] patients sustaining other complications (wound infection, wound dehiscence, nerve injury, tendon injury) at 90d; 34 patients sustained more than one complication within the timeframe.

* Values suppressed according to disclosure control rules as given by NHS Digital (https://digital.nhs.uk/data-and-information/data-tools-and-services/data-services/hospital-episode-statistics/users-uses-and-access-to-hospital-episode-statistics)

Supplementary Table 6. Subhazard ratio (SHR) for risk of revision surgery; crude and adjusted hazard ratio (HR) for risk of other complications

		**Revision Surgery**		**Other Complications**[a]	
		Crude SHR[b] (95%CI)	Adjusted SHR[b] (95%CI)	Crude HR[c] (95%CI)	Adjusted HR[c] (95%CI)
**Sex**	Female	*1 (reference)*	*1*	*1*	*1*
	Male	1·11 (1·07 to 1·15)	1·09 (1·06 to 1·13)	2·30 (1·73 to 3·06)	2·32 (1·74 to 3·09)
**Age group (years)**	18-29	0·83 (0·74 to 0·93)	0·85 (0·76 to 0·96)	2·09 (1·02 to 4·25)	2·25 (1·10 to 4·62)
	30-39	0·96 (0·90 to 1·02)	0·99 (0·93 to 1·05)	1·19 (0·72 to 1·94)	1·20 (0·72 to 2·00)
	40-49	1·08 (1·03 to 1·13)	1·09 (1·04 to 1·14)	0·96 (0·62 to 1·47)	0·98 (0·63 to 1·51)
	50-59	*1 (reference)*	*1*	*1*	*1*
	60-69	1·09 (1·04 to 1·14)	1·05 (1·00 to 1·10)	1·08 (0·70 to 1·67)	0·99 (0·64 to 1·53)
	70-79	1·13 (1·08 to 1·18)	1·06 (1·01 to 1·12)	0·96 (0·61 to 1·53)	0·84 (0·52 to 1·36)
	Over 80	1·16 (1·10 to 1·23)	1·09 (1·03 to 1·15)	0·64 (0·34 to 1·18)	0·59 (0·31 to 1·11)
**Charlson Index**	0	*1 (reference)*	*1*	*1*	*1*
	1	1·21 (1·17 to 1·26)	1·22 (1·18 to 1·28)	0·91 (0·62 to 1·33)	0·98 (0·67 to 1·44)
	2	1·15 (1·09 to 1·20)	1.17 (1·11 to 1·23)	0·94 (0·58 to 1·51)	1·06 (0·65 to 1·73)
	3-4	1·25 (1·19 to 1·31)	1.26 (1·20 to 1·33)	0·75 (0·45 to 1·27)	0·86 (0·50 to 1·48)
	5+	1·22 (1·17 to 1·28)	1.25 (1·19 to 1·32)	1.32 (0·86 to 2·03)	1.48 (0·31 to 1·11)
**Indices of Multiple Deprivation (deciles)**	Least deprived 10%	*1 (reference)*	*1*	1	1
	Less deprived 10-20%	1·03 (0·96 to 1·11)	1·03 (0·96 to 1·11)	0·57 (0·27 to 1·17)	0·55 (0·27 to 1·14)
	Less deprived 20-30%	1·06 (0·99 to 1·14)	1·06 (0·99 to 1·14)	1·01 (0·55 to 1·87)	0·99 (0·53 to 1·83)
	Less deprived 30-40%	1·09 (1·02 to 1·17)	1·09 (1·02 to 1·17)	1·06 (0·58 to 1·94)	1·03 (0·57 to 1·89)
	Less deprived 40-50%	1·07 (1·00 to 1·14)	1·07 (1·00 to 1·15)	1·03 (0·56 to 1·89)	1·00 (0·55 to 1·84)
	More deprived 10-20%	1·20 (1·12 to 1·29)	1·20 (1·12 to 1·29)	1·10 (0·60 to 2·04)	1·05 (0·57 to 1·95)
	More deprived 20-30%	1·15 (1·07 to 1·23)	1·14 (1·05 to 1·20)	0·78 (0·40 to 1·52)	0·75 (0·38 to 1·46)
	More deprived 30-40%	1·13 (1·05 to 1·21)	1·12 (1·05 to 1·20)	0·85 (0·45 to 1·62)	0·82 (0·43 to 1·56)
	More deprived 40-50%	1·11 (1·04 to 1·19)	1·10 (1·03 to 1·18)	0·99 (0·54 to 1·83)	0·96 (0·52 to 1·77)
	Most deprived 10%	1·19 (1·11 to 1·27)	1·18 (1·10 to 1·27)	0·69 (0·34 to 1·39)	0·65 (0·32 to 1·33)

[a]other complications (wound infection, wound dehiscence, nerve injury, tendon injury) at 90 days
[b]Fine and Gray model of competing risks
[c] Cox proportional hazards model

Supplementary Figure 1. Forest plot of adjusted subhazard ratios for reoperation at any time post operatively, accounting for the competing risk of mortality.

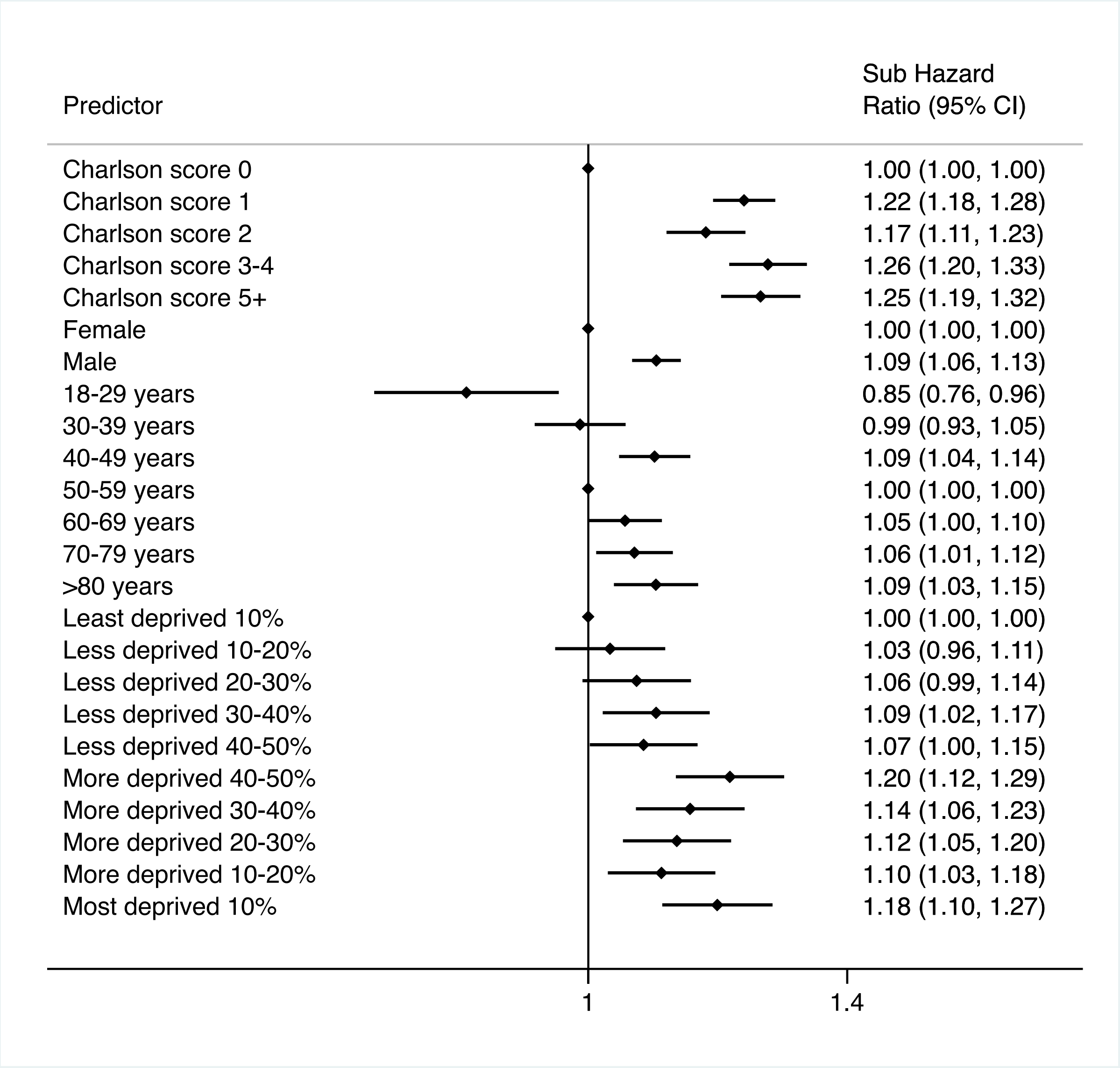

Supplementary Figure 2. Forest plot of adjusted hazard ratios for local complications within 90 days of surgery.

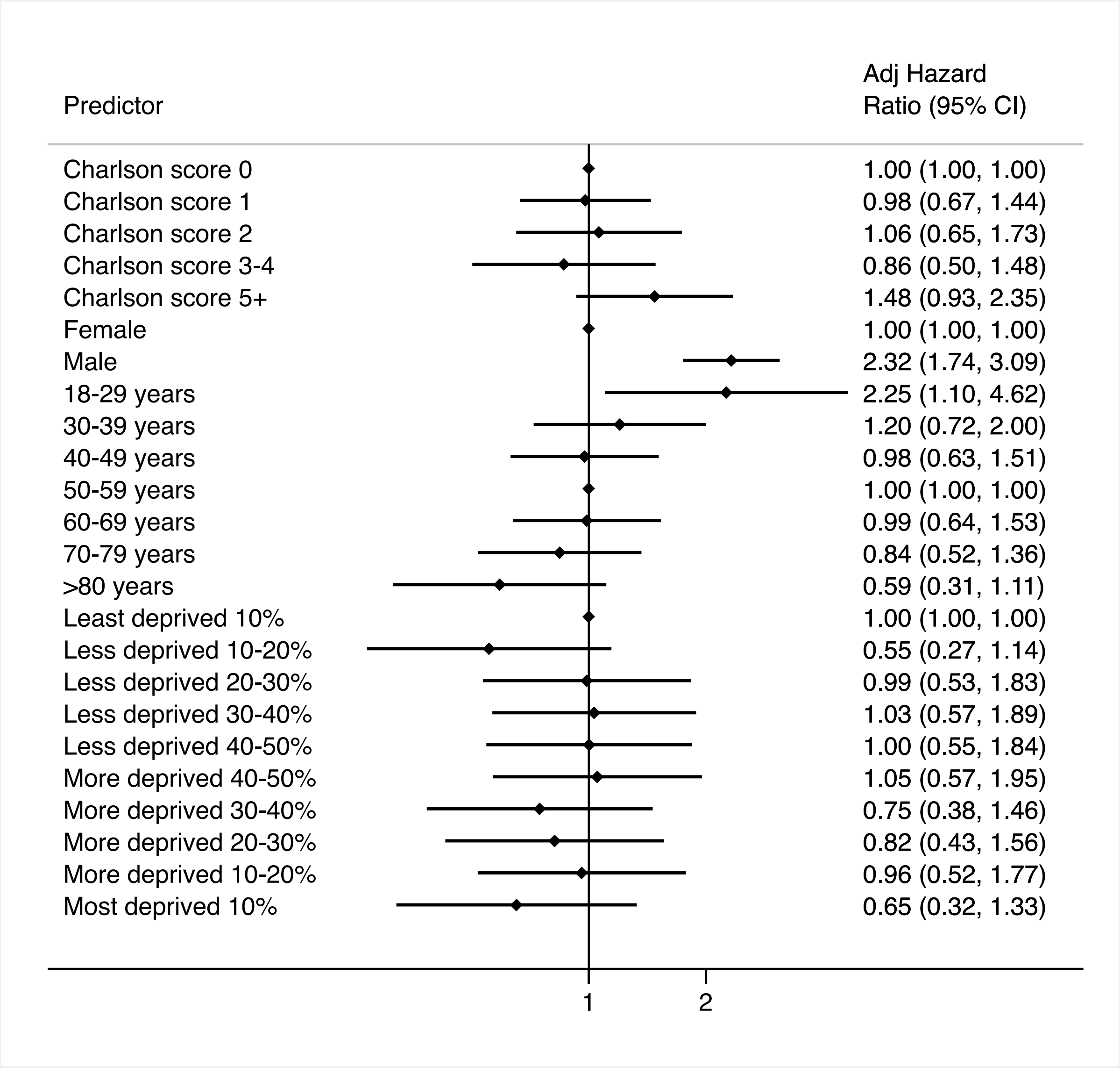

Supplement: Supplementary appendix [file mmc1.pdf]
